# Gender-affirming hormone therapy in cystic fibrosis – A case of new Pseudomonas infection

**DOI:** 10.1016/j.rmcr.2021.101353

**Published:** 2021-01-21

**Authors:** L. Shaffer, K. Bozkanat, M. Lau, P. Sharma, M. Sathe, X. Lopez, R. Jain

**Affiliations:** a University of Texas Southwestern Medical Center, Dallas, TX, USA; b Department of Pediatrics, University of Texas Southwestern Medical Center, Dallas, TX, USA; c Department of Internal Medicine, University of Texas Southwestern Medical Center, Dallas, TX, USA

**Keywords:** Cystic fibrosis, Transgender, Estrogen, *Pseudomonas aeruginosa*

## Abstract

**Background:**

Little is known about the impact of hormone therapy on transgender youth with Cystic Fibrosis (CF). This case report describes an 18-year-old affirmed female with CF who was treated with hormone therapy associated in timing with new growth of *Pseudomonas aeruginosa* in her sputum culture.

**Discussion:**

We highlight important considerations, including the impact of gender-affirming hormone therapy on overall CF disease course. Evidence supports that females with CF have worse outcomes than males, which are partly attributed to estrogen effects. *Pseudomonas aeruginosa* is one of the most prevalent pathogens in people with CF. Here, we highlight a transfemale who grows *Pseudomonas aeruginosa* for the first time since her youth, nearly 1 year after starting estrogen therapy. This is consistent with previous literature of an association between high estrogen levels and *Pseudomonas aeruginosa* prevalence, but has never been evaluated in a transgender population.

**Conclusion:**

Through this case, we demonstrate the need for additional research to understand the relationship between gender-affirmative hormone transition and CF care and management.

## 1 Introduction

Approximately 0.3–0.6% of the world's population identify as transgender and gender diverse (TGD) [1,2], but no published prevalence data in people with Cystic Fibrosis (CF) exist. Little is known about the impact of gender-affirming care, which can include hormone therapy, on the health of people with CF. This case report describes a young adult with CF whose sex assigned at birth was male and identifies as female (affirmed female). This is the first known report in the literature discussing the health implications, specifically host response to bacterial infections in an affirmed female with CF who underwent gender-affirming hormone therapy.

## 2 Case

Our patient is an 18-year-old affirmed female with CF (F508del/F508del) complicated by mild obstructive lung disease (median baseline absolute FEV1 in the 24 months prior to gender transition was 3.40 L (72% using male gender)), chronic sinusitis, and pancreatic insufficiency. She first experienced feelings of gender dysphoria at 12 years of age and slowly began socially transitioning over the next few years. At age 15, she began to see a therapist and psychiatrist at the multidisciplinary specialty clinic for TGD youth. At 16 years old, after discussion with her CF care team, she was started on 1mg of oral 17-β estradiol and 50mg of oral spironolactone (titrated up to 200mg daily) by the adolescent medicine specialist providing gender-affirming care. Two months later, a histrelin acetate implant (gonadotropin-releasing hormone analogue) was subcutaneously placed and spironolactone was discontinued. After five months of puberty suppression and estrogen therapy, the patient's testosterone levels dropped from 719 ng/dL to <5.0 ng/dL (typical baseline range for an adolescent male: 300–1200 ng/dL) and her 17β-estradiol level was 6.20 pg/mL (typical baseline range for pre-menopausal females: 15–350 pg/mL). Seven months later, the dose was increased to 2mg daily of 17β-estradiol which resulted in breast development and a reduction in nocturnal erections, sexual drive, and hair growth/thickness, with her 17β-estradiol level increasing to 34.0 pg/mL. Soon thereafter, the patient was admitted for her first pulmonary exacerbation since undergoing hormone therapy. During that admission, she was also transitioned from CFTR modulator, lumacaftor/ivacaftor to highly effective CFTR modulator, elexacaftor/tezacaftor/ivacaftor, which aided in improving her lung function [3]. After transition to elexacaftor/tezacaftor/ivacaftor, her adolescent medicine subspecialist decreased her 17β-estradiol to 1 mg, which resulted in an estradiol level of 17.0 pg/mL. The patient reported adherence with her hormone and modulator therapy throughout this time.

**Fig. 1:**
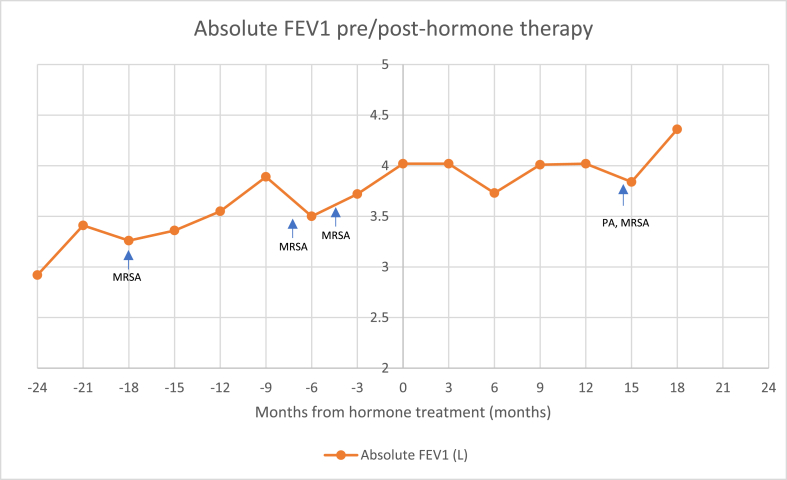
Absolute FEV1 compared to months pre-treatment and months post-treatment with marked culture data. MRSA = Methicillin-resistant *Staph aureus* PA = *Pseudomonas aeruginosa*.

In general, the patient's pulmonary function remained steady since beginning hormonal transition (Fig. 1). However, sixteen months following initiation of hormone therapy, she had her first pulmonary exacerbation requiring hospitalization. Importantly, while she had previously only grown Methicillin-resistant *Staphylococcus aureus* (MRSA) in her sputum cultures, she newly grew *Pseudomonas aeruginosa* (PA) this hospitalization (previously only grown 1 time as a toddler). The identification of PA in her sputum cultures resulted in alteration of her antibiotic regimen to target this organism as part of her exacerbation treatment given that *PA* is a pathogenic bacteria in CF that commonly leads to a progressive decline in lung function and outcomes [4].

## 3 Discussion

This case report highlights the intersection of CF and gender-affirming hormonal therapy. It elucidates several important considerations for care of this unique population, particularly in the context of sex hormones and host response to bacteria.

The implications of gender-affirming hormone therapy on lung health must be considered. Studies show that CF females have worse outcomes and ultimately lower life expectancy compared to CF males [5]. This has, in part, been explained by the effects of sex hormones, particularly estrogen, on mucociliary clearance and the innate immune response [6,7]. Studies in animal models demonstrate that 17β-estradiol can decrease the air surface liquid layer on epithelial cells resulting in worsening mucociliary clearance and cause increased inflammation in response to infection [6,8,9]. Females with CF are more prone to acquire PA infections at an earlier age, resulting in a more rapid decline in lung function following colonization [10]. PA is one of the most common bacteria present in sputum cultures of people with CF [11]. The prevalence is declining overall, particulary in children, but continues to impact 40–60% of individuals [11]. Investigators have also shown that estradiol and estriol are more likely to induce conversion of PA from a non-mucoid to potentially more virulent mucoid phenotype and similarly, high estradiol levels correlate with increased exacerbations with mucoid PA, which is known to have detrimental effects on lung health [12]. Thus, with the initiation of 17β-estradiol therapy and the puberty-suppressant effects of the histrelin acetate implant in this patient, we must be mindful of the potential impact on the trajectory of her disease course as it relates to CF.

It is important to consider the significance of the change in our patient's sputum cultures during her first exacerbation 16 months after initiating hormone therapy. Outside of her toddler years, this was the first instance to grow PA. The timing of recurrence of PA may have been coincidental, but it cannot go unnoticed that this pathogen was cultured during a pulmonary exacerbation while on estrogen therapy, which has been observed in numerous studies to affect women with CF in advance of men [8,12].

A multi-disciplinary approach to care for TGD individuals aimed at providing comprehensive care from providers with various expertise is essential. Our patient had access to physicians specialized in gender-affirming hormone therapy, mental health services, and social work support, along with support from her CF care team. The multidisciplinary nature of the care team ensured the best outcomes for this patient during her gender-affirmation process.

## 4 Conclusion

While the prevalence is unknown, CF individuals who identify as TGD are an important and potentially vulnerable group. This case report demonstrates opportunities for further research to investigate the complex relationship between gender-affirmative care and CF management. These results indicate the importance of collaborative care in a patient's journey through gender transition and highlights potential immune implications based on a host of literature suggesting estrogen may impact host and pathogen response in CF. Future long-term studies are needed to gather additional data on changes in lung function, exacerbation rate, microbiology, inflammation, weight, and mental health that may occur during medical transition. Ultimately, this case report highlights the need for additional research in this field to better understand the impact of gender-affirmation therapy on the health of an individual with CF.

## Declaration of competing interest

The authors report no COI related to this work. R.J receives consulting fees from Vertex Pharmaceuticals and Gilead Sciences unrelated to this work. M.S. receives consulting fees from Alcresta Therapeutics and PBM BC Holdings.
